# Right Upper Canine Tooth in the Left Orbit

**Published:** 1887-07

**Authors:** 


### SELECTIONS.

### Right Upper Canine Tooth in the Left Orbit.

Dr. John Ward Cousins reports, in the *British Medical Journal*, April 23, 1887, a very interesting case of a child, two years old, from whom he removed a tumor of the left orbit, which proved to be a supernumerary and misplaced right upper canine tooth. The crown of the tooth was enclosed in a sac, and the root was attached to the orbital plate by fibro-cartilage. On examination the teeth in the mouth were found in normal position, complete in number, and well formed. The jaws were also large and fully developed for a child two years old.

Irregularity in the number of teeth may occur in either the first or second dentition, and the supernumerary teeth may spring up in any part of the dental arch. In shape these teeth are generally irregular and connical, and they bear no special resemblance to any kind of normal teeth. Sometimes, however, they present a definite outline and accurately resemble one of the recognized forms. Instances of supernumerary incisors, canines, and bicuspids have been recorded, but examples of ill-shaped teeth, without possessing the characters of any special form, are of frequent occurence in dental practice. As regards the time of eruption, supernumerary teeth are always irregular. They are generally matured long before the appearance of the permanent set, but their exact relation to the normal teeth is not well defined. They may be freaks of development in connection with either the temporary or permanent teeth. A normal permanent and a supernumerary tooth sometimes seem to hold the same relation to each other as the teeth of the first and second dentition. In the case of Dr. Cousins there was no irregularity of the teeth, but the age of the patient, and also the special character of the tooth, clearly indicate its relation to the deciduous set.

Cases of misplaced teeth, in strange situations, have often been recorded. These irregularities, however, are not associated with any special shape of jaw, or deficiency of size in the dental arch. The teeth are generally found to occupy an inverted position on the bone to which they are attached. They have been erupted in the hard palate and in the nares, but Dr. Cousins has been unable to find an instance on record of a tooth appearing in the orbit. The occurence of a *right* upper canine in the *left* orbit is certainly singular, and this crossed displacement must have taken place at a very early stage of embryonic life. The canine papillæ appear in the primitive dental groove about the eighth week; and soon after the rudimentary pulps of the milk teeth are in rapid formation within their follicles on the edge of the jaw. At this period, a supernumerary follicle and its contents could be very readily displaced from the lip of the primitive groove, as the surrounding tissues are soft, and the rudimentary orbit and the gums are in close proximity. A supernumerary tooth-sac must always be especially liable to dislocation; and when once it gets out of the groove it may be pushed in any direction during the formation of the surrounding structures.

